# Trends and Patterns of Disparities in Burden of Lung Cancer in the United States, 1974-2015

**DOI:** 10.3389/fonc.2019.00404

**Published:** 2019-05-31

**Authors:** Yu Jie Zhong, Yi Feng Wen, Hai Ming Wong, Guosheng Yin, Ruitao Lin, Shuan Ying Yang

**Affiliations:** ^1^Department of Pulmonary and Critical Care Medicine, The Second Affiliated Hospital of Xi'an Jiaotong University, Xi'an, China; ^2^Key Laboratory of Shaanxi Province for Craniofacial Precision Medicine Research, College of Stomatology, Xi'an Jiaotong University, Xi'an, China; ^3^Public Health and Healthy Ageing Research Group, The University of Hong Kong, Hong Kong, Hong Kong; ^4^Department of Statistics and Actuarial Science, The University of Hong Kong, Hong Kong, Hong Kong; ^5^Department of Biostatistics, The University of Texas MD Anderson Cancer Center, Houston, TX, United States

**Keywords:** lung cancer, trend, disparity, incidence, mortality

## Abstract

**Background:** Although lung cancer incidence and mortality have been declining since the 1990s, the extent to which such progress has been made is unequal across population segments. Updated epidemiologic data on trends and patterns of disparities are lacking.

**Methods:** Data on lung cancer cases and deaths during 1974 to 2015 were extracted from the Surveillance, Epidemiology, and End Results program. Age-standardized lung cancer incidence and mortality and their annual percent changes were calculated by histologic types, demographic variables, and tumor characteristics.

**Results:** Lung cancer incidence decreased since 1990 (1990 to 2007: annual percent change, −0.9 [95% CI, −1.0%, −0.8%]; 2007 to 2015: −2.6 [−2.9%, −2.2%]). Among adults aged between 20 and 39 years, a higher incidence was observed among females during 1995 to 2011, after which a faster decline in female lung cancer incidence (males: −2.5% [−2.8%, −2.2%]; females: −3.1% [−4.7%, −1.5%]) resulted in a lower incidence among females. The white population had a higher incidence than the Black population for small cell carcinoma since 1987. Black females were the only group whose adenocarcinoma incidence plateaued since 2012 (−5.0% [−13.0%, 3.7%]). A higher incidence for squamous cell carcinoma was observed among Black males and females than among white males and females during 1974 to 2015. After circa 2005, octogenarians and older patients constituted the group with the highest lung cancer incidence. Incidence for localized and AJCC/TNM stage I lung cancer among octogenarians and older patients plateaued since 2009, while mortality continued to rise (localized: 1.4% [0.6%, 2.1%]; stage I: 6.7% [4.5%, 9.0%]).

**Conclusions:** Lung cancer disparities prevail across population segments. Our findings inform effective approaches to eliminate lung cancer disparities by targeting at-risk populations.

## Introduction

As of 2018, lung cancer remains the leading cause of cancer incidence and mortality worldwide (1). In the United States, lung cancer is the most common cancer affecting both sexes and is expected to account for more deaths than breast, prostate, and colon cancer combined (2). Cigarette smoking accounts for 81.7% of lung cancer cases and 81.3% of lung cancer deaths (3). Since the release of the first Surgeon General's report in 1964, the American Cancer Society has implemented a range of tobacco-control campaigns with support from the National Cancer Institute. This resulted in a steady decrease in lung cancer incidence overall and a diminishing gender gap (4). However, the extent to which such progress has been made is unequal across various histologic types and across all segments of the population.

Despite an overall reduction in lung cancer incidence, recent studies of national data revealed an increased incidence for squamous cell carcinoma from 2005 to 2010 among females but not among males (5). In contrast, the adenocarcinoma incidence rate during the same period increased among every sex-by-race group (6). In addition to sex and race, disparities in the treatment provided to old and young patients also exist. Lung cancer occurs disproportionally among the elderly population (7). However, they are underrepresented in landmark clinical trials (e.g., National Lung Screening Trial (8), Radiation Therapy Oncology Group (RTOG) 9410 trial (9), and RTOG 0671 trial (10)), resulting in paucity of information on efficacy and safety profiles of various regimens among the elderly (11). The situation is not better among real-world patients. A population-based study showed that only 25.8% of the elderly, aged above 65 years, received first-line chemotherapy (12).

The epidemiological profile of lung cancer is ever-evolving. Available data on trends and disparities of lung cancer are dated (5, 6, 13–15). In this updated and expanded analysis of the most recent data available from the SEER database, we sought to characterize the trends in the lung cancer epidemic over the past 40 years, and to examine patterns of disparities in the burden of lung cancer among different segments of the US population. This study is valuable in formulating targeted approaches that address American Cancer Society's goal to eliminate cancer disparity.

## Methods

### Data Sources

The present study was performed in full compliance with the National Cancer Institute's SEER limited-use data agreement. Per Xi'an Jiaotong University policy, the present study was exempt from approval of the institutional review board because all data used had been deidentified. No patient informed consent was directly obtained. Data analysis took place from July 5, 2018 to December 15, 2018.

Lung cancer cases diagnosed during 1974 to 2015 were abstracted from the SEER 9 research database (16). The database includes information from nine long standing, high-quality, population-based registries (California [San Francisco and Oakland], Connecticut, Georgia [Atlanta only], Hawaii, Iowa, Michigan [Detroit only], New Mexico, Utah, and Washington [Seattle and Puget Sound region]) which represent ~10% of the total population in the US. Information on demographics and cancer diagnoses were available for each case in the database.

Observed nationwide and SEER 9 lung cancer deaths were retrieved from information recorded in death certificates and were ascertained from the National Center for Health Statistics (17). To examine lung cancer mortality according to tumor characteristics at diagnosis, incidence-based mortality rates were estimated from the SEER 9 cancer incidence file that linked cancer characteristics at diagnosis with death certificate information (18, 19). We allowed for 15 years of follow-up to ensure that the incidence-based mortality rates accurately reflected observed death certificate mortality rates (18). Therefore, the estimated incidence-based mortality rates were confined to cases of death during 1989 to 2015 that had been diagnosed during 1974 to 2015. Incidence and mortality rates reported in this study represent the most recent follow-ups available in the SEER database.

### Demographic and Tumor Characteristics

Demographic variables including age, sex, and race were obtained from the registry databases. Three major histologic types of lung cancer (*International Classification of Diseases for Oncology, Third Edition*; topography code C34) were classified according to a morphology code: small cell carcinoma (8041–8045), adenocarcinoma (8140, 8211, 8230–8231, 8250–8260, 8323, 8480–8490, 8550–8551, 8570–8574, 8576), and squamous cell carcinoma (8050–8078, 8083–8084).

The SEER Historic Stage A definition of the extent of the disease at diagnosis, available for lung cancer since 1988, was used to classify cancer stages into localized, regional, distant, and unknown stages (20). The American Joint Committee on Cancer/Union for International Cancer Control (AJCC/UICC) tumor-node-metastasis (TNM) staging system (*AJCC Cancer Staging Manual, 6th Edition*) was incorporated into the SEER database from 2004 onward and was used to classify cases into stages I-IV (or unknown) (21). We included the AJCC/TNM staging system in our analysis since it is more clinically relevant than the SEER stage.

### Data Analysis

Patients were included only when the cases had been microscopically confirmed. Only the first matching record of each patient was used for patient selection. Patients whose deaths had been reported by autopsy only, or by death certificate only, were excluded. Only malignant cases (*International Classification of Diseases for Oncology, Third Edition*, behavior code of /3) were included. All incidence and mortality rates were age-standardized to the 2,000 US standard population and expressed per 100,000 population. Incidence-based mortality rates were estimated as the number of lung cancer deaths among incident cases diagnosed in the SEER 9 registries over a total person-time at risk in the same area. SEER^*^Stat software (version 8.3.5) was used to calculate all rates (22). Lung cancer incidence from 1974 to 2015 and incidence-based mortality from 1989 to 2015 were calculated according to demographic variables (age, sex, and race) and tumor characteristics (SEER stage and AJCC/TNM stage) for all histologic types combined, and three major histologic types. Disparities in the burden of lung cancer among subpopulations with various demographic variables and tumor characteristics were examined by histologic type. The numbers of cases were described with absolute and relative frequencies, and rates were described with means and Tiwari modified confidence intervals (CIs) (23).

To characterize patterns of trends in lung cancer incidence and mortality rates, the joinpoint regression model was applied to estimate annual percent changes (APCs) for consecutive calendar years with similar secular trends in rates. The years in which statistically significant changes in APCs occurred were designated as the joinpoints. The average of the APCs weighted by their corresponding time interval lengths yielded the average annual percent changes (AAPCs), which was a summary measure of the trend over the entire study period. The National Cancer Institute's Joinpoint Regression Program (version 4.6.0.0) was used for the joinpoint regression analyses (24). The optimal number of joinpoints was determined through permutation tests. Statistical significance of the APCs and AAPCs was evaluated through the method proposed by Fay et al. (25) Temporal trends in rates were plotted against years with joinpoint regression lines added to the graph. Semi-logarithmic scales with a y- to x-axis ratio of one logarithmic cycle equal to the length of 40 years were used for plotting such that a slope of 10 degrees for the regression lines corresponded to 1% annual rate change (26). Figures were created using the tidyverse package, version 1.2.1 (27), on R, version 3.5.1 R Development Core Team (28). The level of statistical significance was set at 0.05 and all tests were two-sided.

## Results

Of the 618,226 lung cancer cases diagnosed among residents of the SEER-9 areas during 1975 to 2015, 537,719 (87.0%) met the case definition and were eligible for analysis of lung cancer incidence (Table 1). Males (absolute frequency [relative frequency], 316,804 [58.9%]) and elderly patients aged 60 or above (408,099 [75.9%]) comprised the majority of the cases. Although Black patients constituted only 10.6% of the cases, their incidence rate was higher than that of the white population. Adenocarcinoma and squamous cell carcinoma occurred in 60.2% of the cases. Among the eligible cases, 278,812 died of lung cancer during 1989 to 2015 and were included for estimation of incidence-based mortality. Of these 278,812 deaths, 56.8% were males and 78.0% were aged 60 or above.

**Table 1:** Lung cancer incidence (1974–2015): The SEER-9 registry database.

**Characteristic**	**Overall**		**Small cell carcinoma**		**Adenocarcinoma**		**Squamous cell carcinoma**	
	**Cases, No. (%)^a^**	**Rate (95% CI)^b^**	**Cases, No. (%)^a^**	**Rate (95% CI)^b^**	**Cases, No. (%)^a^**	**Rate (95% CI)^b^**	**Cases, No. (%)^a^**	**Rate (95% CI)^b^**
Overall	537,719 (100)	53.47 (53.33–53.61)	83,403 (100)	8.22 (8.16-8.27)	191,577 (100)	19.05 (18.97–19.14)	132,074 (100)	13.17(13.10–13.24)
**Sex**								
Male	316,804 (58.9)	71.79 (71.54–72.05)	46,251 (55.5)	10.28 (10.18–10.37)	100,078 (52.2)	22.61 (22.47–22.76)	93,045 (70.4)	21.25 (21.11–21.39)
Female	220,915 (41.1)	39.71 (39.54–39.88)	37,152 (44.5)	6.66 (6.59–6.73)	91,499 (47.8)	16.47 (16.36–16.57)	39,029 (29.6)	7.00 (6.93–7.07)
**Race**								
White	448,127 (83.3)	53.63 (53.47–53.79)	73,290 (87.9)	8.70 (8.64–8.76)	156,860 (81.9)	18.79 (18.70–18.88)	109,685 (83.0)	13.13 (13.06–13.21)
Black	56,861 (10.6)	67.46 (66.90–68.04)	6,542 (7.9)	7.75 (7.56–7.94)	19,649 (10.2)	22.95 (22.62–23.28)	15,917 (12.1)	19.44 (19.13–19.75)
Other^c^	32,731 (6.1)	37.93 (37.52–38.35)	3,571 (4.3)	4.07 (3.94–4.21)	15,068 (7.9)	17.42 (17.14–17.70)	6,472 (4.9)	7.58 (7.40–7.77)
**Age at diagnosis**								
<20	165 (0.0)	0.05 (0.05–0.06)	NR^d^	NR^d^	NR^d^	NR^d^	NR^d^	NR^d^
20–39	5,156 (1.0)	1.76 (1.71–1.81)	422 (0.5)	0.15 (0.13–0.16)	2,081 (1.1)	0.72 (0.69–0.75)	556 (0.4)	0.19 (0.18–0.21)
40–59	124,299 (23.1)	45.40 (45.15–45.66)	20,249 (24.3)	7.35 (7.25–7.45)	47,746 (24.9)	17.50 (17.35–17.66)	24,552 (18.6)	8.89 (8.78–9.00)
60–79	339,140 (63.1)	253.97 (253.11–254.84)	54,503 (65.3)	40.48 (40.14–40.83)	116,493 (60.8)	87.12 (86.62–87.63)	89,600 (67.8)	67.42 (66.98–67.87)
≥80	68,959 (12.8)	223.02 (221.36–224.69)	8,226 (9.9)	26.63 (26.06–27.21)	25,251 (13.2)	81.62 (80.62–82.64)	17,360 (13.2)	56.18 (55.34–57.02)
**LUNG CANCER CHARACTERISTICS AT DIAGNOSIS**								
**SEER histologic stage A^e^**								
Localized	73,580 (18.6)	10.01 (9.94–10.08)	3,715 (6.3)	0.50 (0.49–0.52)	33,996 (22.4)	4.60 (4.56–4.65)	20,453 (23.3)	2.81 (2.77–2.85)
Regional	100,901 (25.5)	13.62 (13.53–13.70)	13,168 (22.2)	1.76 (1.73–1.79)	34,734 (22.9)	4.68 (4.63–4.73)	30,959 (35.3)	4.20 (4.15–4.25)
Distant	202,305 (51.2)	27.26 (27.14–27.38)	39,715 (67.1)	5.33 (5.28–5.38)	77,946 (51.4)	10.49 (10.42–10.57)	31,572 (36.0)	4.28 (4.23–4.33)
Unknown	18,391 (4.7)	2.52 (2.48–2.56)	2,606 (4.4)	0.35 (0.34–0.37)	5,004 (3.3)	0.69 (0.67–0.71)	4,780 (5.4)	0.66 (0.64–0.68)
**AJCC/TNM stage^f^**								
I	34,998 (20.0)	9.88 (9.77–9.98)	1,023 (4.3)	0.29 (0.27–0.31)	19,641 (25.9)	5.50 (5.42–5.57)	9,615 (26.4)	2.76 (2.71–2.82)
II	7,591 (4.3)	2.12 (2.07–2.16)	475 (2.0)	0.13 (0.12–0.14)	3,143 (4.1)	0.87 (0.84–0.90)	2,689 (7.4)	0.75 (0.73–0.78)
III	41,484 (23.6)	11.53 (11.41–11.64)	6,238 (26.3)	1.71 (1.67–1.76)	15,833 (20.9)	4.40 (4.33–4.46)	10,757 (29.5)	3.01 (2.95–3.07)
IV	79,264 (45.2)	21.88 (21.73-22.04)	14,878 (62.6)	4.08 (4.02–4.15)	34,156 (45.0)	9.40 (9.30–9.50)	11,361 (31.3)	3.19 (3.13–3.25)
Unknown	12,157 (6.9)	3.41 (3.35–3.47)	1,138 (4.8)	0.32 (0.30–0.34)	3,115 (4.1)	0.88 (0.85–0.91)	1,981 (5.4)	0.56 (0.54–0.59)

*AJCC/TNM, American Joint Committee on Cancer/ Tumor-Node Metastasis; NR, not reported; SEER, Surveillance, Epidemiology, and End Results*.

a *Cases included first primary tumors that matched the selection criteria, were microscopically confirmed, and were not identified only from autopsy records or death certificates*.

b *Rates were calculated as number of cases per 100,000 person-years and age adjusted to the 2,000 US standard population*.

c *Includes American Indian/Alaskan Native and Asian/Pacific Islander*.

d *Statistic suppressed because of fewer than 16 cases in the time interval*.

e *SEER Histologic Stage A is based on cases diagnosed during 1988–2015*.

f *AJCC/TNM stage is based on cases diagnosed during 2004–2015*.

**Figure 1:**
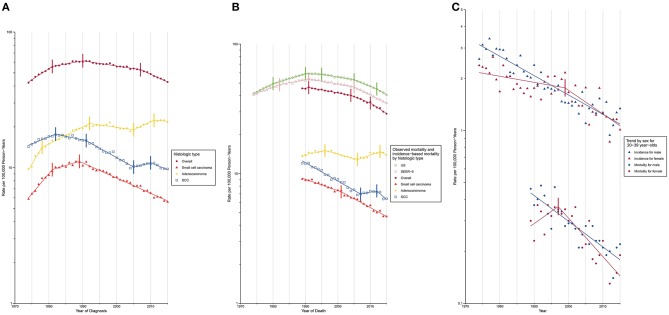
Trends in Annual Lung Cancer Incidence and Mortality Rates. SCC, squamous cell carcinoma. Data markers denote the observed incidence and mortality rates (cases per 100,000 person-years). The slope of the lines represents APC; vertical tics on these lines represent the joinpoints. Rates are age-adjusted to the 2,000 US standard population. **(A)** Overall lung cancer incidence and incidence by histologic type. **(B)** Overall lung cancer mortality and mortality by histologic type. **(C)** Overall lung cancer incidence and mortality among young adults aged 20–39 years.

Trends in lung cancer incidence by demographic variables and tumor characteristics at diagnosis are described in Supplementary Table 1 and are shown graphically in Figure 1A. Lung cancer incidence increased during 1974 to 1981 (APC, 3.8% [95% CI, 3.4%, 4.3%]) and 1981–1990 (APC, 1.1 [0.7%, 1.4%]), followed by a sustained decrease from 1990 to 2007 (APC, −0.9 [−1.0%, −0.8%]) and 2007 to 2015 (APC, −2.6 [−2.9%, −2.2%]). Lung cancer incidence-based mortality rates during 1989 to 2015 were consistent with observed nationwide and SEER 9 mortality rates (Figure 1B; Supplementary Table 2). Lung cancer incidence-based mortality rates (Table 2) decreased, on average, 1.7% (−1.9%, 1.4%) per year from 1989 to 2015, with a continuous decline from 1991 onward.

**Table 2:** Lung cancer incidence-based mortality (1989–2015): The SEER-9 registry database.

**Characteristic**	**Overall**		**Small cell carcinoma**		**Adenocarcinoma**		**Squamous cell carcinoma**	
	**Deaths, No. (%)^a^**	**Rate (95% CI)^b^**	**Deaths, No. (%)^a^**	**Rate (95% CI)^b^**	**Deaths, No. (%)^a^**	**Rate (95% CI)^b^**	**Deaths, No. (%)^a^**	**Rate (95% CI)^b^**
Overall	278,812 (100)	38.97 (38.83–39.12)	48,422 (100)	6.73 (6.67–6.79)	100,340 (100)	14.00 (13.91–14.09)	60,344 (100)	8.51 (8.44–8.58)
**Sex**								
Male	158,238 (56.8)	50.91 (50.66–51.17)	25,153 (51.9)	7.91 (7.81–8.01)	52,486 (52.3)	16.82 (16.68–16.97)	40,300 (66.8)	13.21 (13.08–13.34)
Female	120,574 (43.2)	30.19 (30.01–30.36)	23,269 (48.1)	5.86 (5.79–5.94)	47,854 (47.7)	11.95 (11.84–12.06)	20,044 (33.2)	5.02 (4.95–5.09)
**Race**								
White	230,350 (82.6)	39.38 (39.22–39.54)	42,365 (87.5)	7.21 (7.15–7.28)	81,575 (81.3)	13.93 (13.83–14.02)	49,511 (82.1)	8.50 (8.43–8.58)
Black	30,117 (10.8)	49.11 (48.54–49.68)	3,803 (7.9)	6.20 (6.00–6.41)	10,611 (10.6)	17.01 (16.68–17.34)	7,444 (12.3)	12.71 (12.42–13.01)
Other^c^	18,345 (6.6)	26.30 (25.92–26.69)	2,254 (4.6)	3.19 (3.05–3.32)	8,154 (8.1)	11.66 (11.41–11.92)	3,389 (5.6)	4.93 (4.76–5.10)
**Age at diagnosis**								
<20	NR^d^	NR^d^	NR^d^	NR^d^	NR^d^	NR^d^	NR^d^	NR^d^
20–39	1,980 (0.7)	0.28 (0.27–0.29)	209 (0.4)	0.03 (0.03–0.03)	991 (1.0)	0.14 (0.13–0.15)	204 (0.3)	0.03 (0.03–0.03)
40–59	59,356 (21.3)	7.76 (7.70–7.82)	10,706 (22.1)	1.39 (1.36–1.42)	23,560 (23.5)	3.09 (3.05–3.13)	9,715 (16.1)	1.26 (1.24–1.29)
60–79	175,711 (63.0)	25.04 (24.92–25.16)	31,893 (65.9)	4.51 (4.46–4.56)	60,719 (60.5)	8.65 (8.58–8.72)	40,592 (67.3)	5.83 (5.77–5.88)
≥80	41,755 (15.0)	5.89 (5.83–5.95)	5,613 (11.6)	0.80 (0.78–0.82)	15,069 (15.0)	2.12 (2.09–2.15)	9,830 (16.3)	1.39 (1.36–1.42)
**LUNG CANCER CHARACTERISTICS AT DIAGNOSIS**								
**SEER histologic stage A^e^**								
Localized	29,552 (10.6)	4.21 (4.16–4.26)	2,544 (5.3)	0.36 (0.35–0.37)	11,845 (11.8)	1.68 (1.65–1.71)	9,139 (15.1)	1.31 (1.28–1.34)
Regional	66,522 (23.8)	9.33 (9.25–9.40)	10,032 (20.7)	1.39 (1.37–1.42)	20,674 (20.6)	2.89 (2.85–2.93)	20,519 (34.0)	2.89 (2.85–2.93)
Distant	163,327 (58.6)	22.70 (22.59–22.81)	33,185 (68.5)	4.60 (4.55–4.65)	61,682 (61.5)	8.56 (8.49–8.63)	25,369 (42.1)	3.55 (3.51–3.59)
Unknown	19,411 (7.0)	2.74 (2.70–2.78)	2,661 (5.5)	0.37 (0.36–0.39)	6,139 (6.1)	0.87 (0.84–0.89)	5,317 (8.8)	0.76 (0.74–0.78)
**AJCC/TNM stage^f^**								
I	9,625 (7.8)	2.77 (2.71–2.82)	546 (2.7)	0.16 (0.14–0.17)	4,295 (8.7)	1.22 (1.19–1.26)	3,072 (12.4)	0.90 (0.86–0.93)
II	3,635 (2.9)	1.03 (1.00–2.06)	261 (1.3)	0.07 (0.06–0.08)	1,383 (2.8)	0.39 (0.37–0.41)	1,281 (5.2)	0.37 (0.35–0.39)
III	27,374 (22.0)	7.67 (7.57–7.76)	4,445 (22.2)	1.24 (1.20–1.27)	9,752 (19.7)	2.73 (2.67–2.78)	7,013 (28.3)	1.98 (1.93–2.03)
IV	62,169 (50.1)	17.20 (17.07–17.34)	12,179 (60.8)	3.36 (3.30–3.42)	25,804 (52.2)	7.11 (7.02–7.20)	8,907 (36.0)	2.50 (2.45–2.56)
Unknown	21,373 (17.2)	6.06 (5.98–6.14)	2,594 (13.0)	0.72 (0.70–0.75)	8,209 (16.6)	2.33 (2.28–2.38)	4,475 (18.1)	1.29 (1.25–1.33)

*AJCC/TNM, American Joint Committee on Cancer/ Tumor-Node Metastasis; NR, not reported; SEER, Surveillance, Epidemiology, and End Results*.

a *No. (%) of deaths were based on cases diagnosed during 1974–2015*.

b *Rates were calculated as number of deaths per 100,000 person-years and age adjusted to the 2,000 US standard population*.

c *Includes American Indian/Alaskan Native and Asian/Pacific Islander*.

d *Statistic suppressed because of fewer than 16 deaths in the time interval*.

e *SEER Histologic Stage A is based on cases diagnosed during 1988–2015 and deaths during 1989–2015*.

f *AJCC/TNM stage is based on cases diagnosed during 2004–2015 and deaths during 2004–2015*.

Trends in lung cancer incidence rates and incidence-based mortality rates by demographic variables and tumor characteristics at diagnosis, are graphically depicted in Supplementary Figures 1–40. Lung cancer incidence peaked in males in 1988, while that in females continued to increase until 2006 (Supplementary Figure 1). The gender gap in lung cancer incidence has been narrowing over time. Incidence rate for males was 3.7 times than that for females in 1974 (males, 71.68 [standard error (SE), 1.03]; females, 19.39 [SE, 0.47]), whereas the number dropped to 1.3 in 2015 (males, 48.7 [SE, 0.58]; females, 38.29 [SE, 0.46]). The narrowing gender gap was also evident for lung cancer incidence-based mortality over time (Supplementary Figure 2). A reduction in incidence (Supplementary Figure 17) was observed among the population aged below 60 years (20–39 years: AAPC, −2.1% [−2.4%, −1.9%]; 40–59 years: AAPC, −1.8% [−2.0%, −1.6%]). Among young adults aged between 20 to 39 years, a higher incidence was noted among females compared to males during 1995 to 2011, due to a slower decline in incidence among females prior to 1999 (males: APC, −2.5% [−2.8%, −2.2%]; females: APC, −0.8% [−1.5%, −1.1%]). After 1999, however, a rapid reduction in female lung cancer incidence (males: APC, −2.5% [−2.8%, −2.2%]; females: APC, −3.1% [−4.7%, −1.5%]) resulted in a lower incidence among females from 2011 onward (Figure 1C; Supplementary Table 3). A similar mortality trend was observed among this young population.

**Figure 2:**
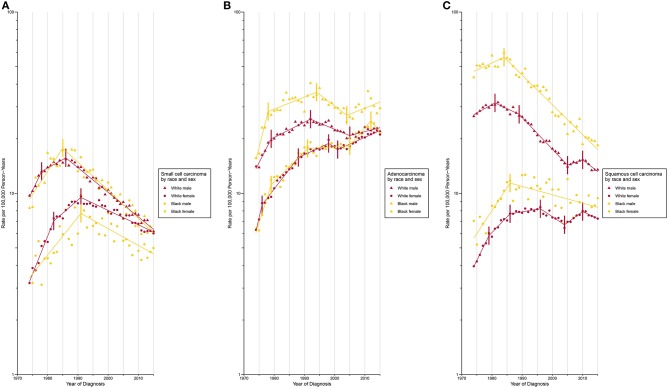
Trends in Annual Lung Cancer Incidence Rates by Sex and Race According to Histologic Type. **(A)** Age-standardized incidence rate for small cell carcinoma. **(B)** Age-standardized incidence rate for adenocarcinoma. **(C)** Age-standardized incidence rate for squamous cell carcinoma.

Incidence for small cell carcinoma decreased significantly (incidence: APC, −2.5% [−2.7%, −2.4%]; mortality: AAPC, −2.6% [−2.8%, −2.4%]) from 1989 onward (Figures 1A,B; Supplementary Tables 1, 2). The white population consistently had a higher incidence than its Black counterpart since 1987 with a slightly widening gap over time (Supplementary Figure 11). When the population was classified by both sex and race, the incidence was lowest among Black females and the rate difference with other sex-by-race groups continued to widen since 1991 (Figure 2A).

Adenocarcinoma is the only histologic type with a non-decreasing incidence (AAPC, 2.0% [1.6%, 2.5%]) and mortality (AAPC, 0.0% [−0.5%, 0.6%]) rate during the study period (Figures 1A,B; Supplementary Tables 1, 2). This resulted in a cross of incidence for adenocarcinoma and squamous cell carcinoma, with adenocarcinoma being the most common type of lung cancer since 1985. Two waves of increase in incidence (Figure 1A; Supplementary Table 1) were observed during 1974 to 1992 and 2005 to 2011 (APC, 2.7% [1.0%, 4.4%]). The most rapid increase occurred from 1974 to 1978 with a change of 10.1% per year, on average. A faster incidence increase was observed among Black females (APC, 4.6% [95% CI, 1.6% to 7.8%]) compared to other sex-by-race groups during 2005 to 2012 (Figure 2B; Supplementary Table 4). Black females were the only group whose incidence ceased to increase since 2012 (APC, −5.0% [−13.0%, 3.7%]).

The incidence (APC, −1.0% [−1.4%, −0.6%]) and mortality (APC, −2.6% [−3.3%, −1.9%]) of squamous cell carcinoma decreased over time (Figures 1A,B; Supplementary Tables 1, 2). A closer examination revealed a consistently higher incidence among Black males and females than among white males and females during the entire study period (Figure 2C).

**Figure 3:**
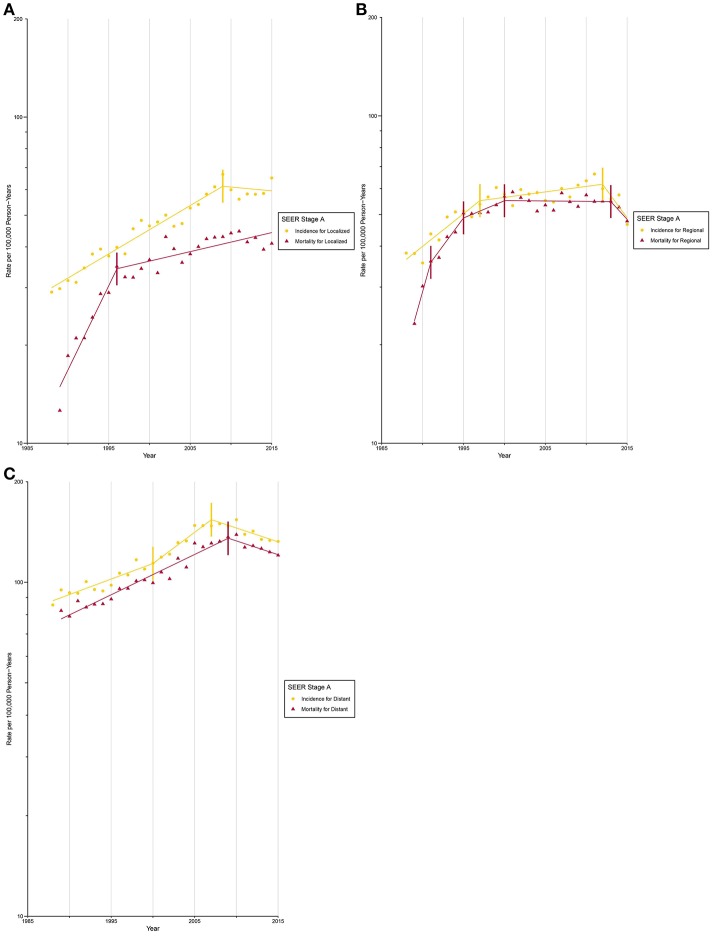
Trends in Annual Lung Cancer Incidence and Mortality Rates among Elderly Aged 80 and above by SEER stage at diagnosis. **(A)** Incidence and mortality rates for localized SEER stage at diagnosis. **(B)** Incidence and mortality rates for regional SEER stage at diagnosis. **(C)** Incidence and mortality rates for distant SEER stage at diagnosis.

**Figure 4:**
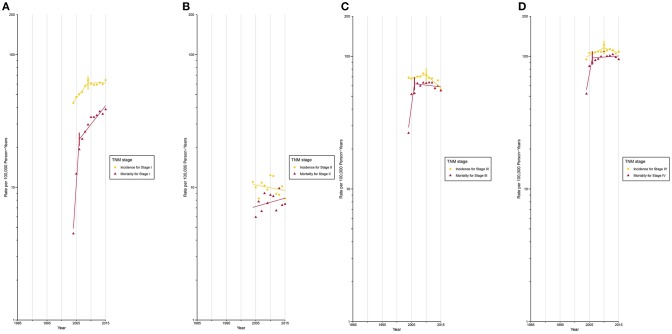
Trends in Annual Lung Cancer Incidence and Mortality Rates among Elderly Aged 80 and above by TNM stage at diagnosis. **(A)** Incidence and mortality rates for TNM stage I at diagnosis. **(B)** Incidence and mortality rates for TNM stage II at diagnosis. **(C)** Incidence and mortality rates for TNM stage III at diagnosis. **(D)** Incidence and mortality rates for TNM stage IV at diagnosis.

After circa 2005, octogenarians and older patients constituted the group with the highest lung cancer incidence rate (Supplementary Figure 17; Supplementary Tables 5, 6). The incidence for localized (Figure 3A) and AJCC/TNM stage I (Figure 4A) lung cancer among octogenarians and older patients plateaued since 2009, while mortality continued to rise rapidly (localized SEER stage: APC, 1.4% [0.6%, 2.1%]; AJCC/TNM stage I: APC, 6.7% [4.5%, 9.0%]). The incidence and mortality for regional and distant SEER stage (Figures 3B,C) and AJCC/TNM stage II-IV (Figures 4B-D) lung cancer were closely aligned.

## Discussion

In this population-based study, we confirmed, updated, and extended previous studies on the epidemiological profile of lung cancer in the US. Consistent with previous results (29), we found a declining lung cancer incidence and mortality beginning from 1990 and 1991, respectively. A faster decline among males resulted in diminishing gender gaps in lung cancer (30). We updated previous trend studies (5, 6, 13–15) and revealed the changing landscape of lung cancer types through demographic variables. We further extended our analysis to examine trends of lung cancer incidence and mortality for octogenarians and older patients, by cancer stage at diagnosis. The mortality rate for those diagnosed with early-stage lung cancer has constantly been rising, even when the incidence rate has plateaued.

The decline in lung cancer incidence among females occurred in 2006, 18 years after the male incidence began to decline in 1988. This has been associated with the later uptake of smoking cessation among females (31), lower female cessation rates (32), and large variations in the level of tobacco control across states in the US (33). Convergence in smoking behaviors between males and females led to the diminishing gender gap in lung cancer mortality, which is expected to close circa 2,045 (34). The trend of lung cancer among young adults is a sentinel measure of the effectiveness of tobacco control programs (35). Although the trend among the young population is unlikely to substantially affect the status quo epidemiological profile of lung cancer, due to its relatively small fraction, it could considerably impact the lung cancer burden and other smoking-related diseases 30 years later, as this cohort ages (34).

In line with a recent report by Jemal et al. (36) we observed a higher lung cancer incidence among young females than males during 1995 to 2011 among 20 to 39-year-olds. Unlike the report by Jemal et al., however, we observed a re-emergence of male preponderance after 2011. A faster decline in lung cancer incidence in young females from 1999 onwards explained the changing landscape of gender preponderance. Differences between our findings and that of Jemal et al., deserve further investigation. One possible explanation may be our inclusion of younger adults aged between 20 to 29 years. Another possibility is the effect of etiologic factors other than smoking, which evolved to be more important in the contemporary birth cohort whose smoking prevalence is historically low, which differentially affects young males and females. Trends for mortality were generally reminiscent of trends for incidence. The non-decreasing mortality during 1989 to 1997 could be partially explained by an increased initiation of smoking among girls born after circa 1950 (37), during whose adolescent and early adulthood period cigarette brands such as Virginia Slims were aggressively marketed to women (38–40).

Gaps in lung cancer rates narrowed between the Black and white population due to a greater reduction in smoking initiation among the Black population since the 1970s (41). A lower incidence of small cell carcinoma among Black females, the group with the lowest smoking prevalence among the sex-by-race groups investigated (42), is consistent with the notion that small cell carcinoma is intimately associated with smoking (43), However, higher incidence among the white population than among the Black population, as reported previously (5), albeit the white populations' lower smoking prevalence (42), highlights the biological complexity and tremendous need for etiologic elucidation of this “recalcitrant cancer.”

Historically, the first wave of increase in adenocarcinoma during 1974 to 1992 was partially influenced by the introduction of novel diagnostic methods that improved access to lung periphery, where adenocarcinoma predominantly develops (14). The second wave of increase was related to changes in cigarette composition and design (44, 45) and more accurate histologic classification (46). Tapering of adenocarcinoma among Black females was reported for the first time since its universal rise beginning over a decade ago. Continued surveillance that substantiates the observed trends and explores the relative contribution of smoking and other etiologic factors is warranted, to inform evidence-based strategies targeting at-risk populations, to effectively reduce the burden associated with this dominant type of lung cancer.

Incidence for squamous cell carcinoma decreased steadily since the 1980s. A consistently higher incidence was noted among Black males and females compared to white males and females. Squamous cell carcinoma is more strongly associated with smoking than other histologic types (47, 48). Despite the lower initiation rate and fewer cigarettes smoked (41), the Black population had a lower cessation rate, partly explained by the aggressive advertisement of mentholated cigarettes to the Black population (49). Targeted advertisement (49), higher neighborhood retailer density (50), and inadequate exposure to tobacco control welfare services (50) result in racial disparities and lung cancer becoming a social justice issue.

With the population aging in the US, the burden of lung cancer is increasingly concentrated among the vulnerable elderly. The close alignment of incidence and mortality trends among octogenarians and older patients diagnosed at an advanced stage, reflects a limited treatment improvement. More disconcerting are the findings for those diagnosed at earlier stages. A continued increase in mortality, when incidence has plateaued, provides circumstantial evidence that the elderly are undertreated (51). In the era of precision medicine, there is an urgent need to increase the evidence base for treatment of the elderly with varying fitness levels, to formulate precise clinical guidelines for each subgroup, and to reduce therapeutic nihilism on both the patients' and clinicians' side to strengthen the implementation of these guidelines.

The present study has significant clinical implications. With decreasing rates of types of lung cancer which are evidently associated with smoking, more research efforts are expected to elucidate etiologic factors of other types of lung cancer, particularly adenocarcinoma. In addition, the novel changes in the epidemiological profile of lung cancer revealed in this study, highlight prevailing disparities in the burden of lung cancer across population segments. Our findings are of value in informing polices that aim to achieve the continued reduction of lung cancer burden and to promote social equity. This study had several limitations. First, the study is descriptive in nature and we were unable to rule out the possibility of ecological fallacy (52). Second, data on smoking behavior, socioeconomic status, and comorbidities had not been captured by the SEER database and thus were not adjusted for in our study. Third, inaccuracies in cause of death from death certificates might compromise interpretation of mortality trends. Fourth, SEER oversamples urban and foreign-born populations, which might limit the generalizability of study findings. However, the high confirmation rate of lung cancer in the US (53) and the consistency of incidence-based mortality with observed nationwide and SEER 9 lung cancer death rates suggest these drawbacks are unlikely to substantially bias the study findings. Access to health care is an important factor associated with both the incidence and mortality of lung cancer. Although the present study provides solid evidence on disparities of the burden of lung cancer across population segments in the US, factors contributing to such disparities remain elusive. The effect of limited access to adequate health care among the vulnerable population on disparities of lung cancer warrants further research investigation.

Lung cancer incidence and mortality declined overall in the US during 1974 to 2015 with diminishing gender gaps. However, disparities in the lung cancer burden still prevail across segments of the population. Unmet health care needs among the already vulnerable and frail octogenarians and older lung cancer patients are devastating from a medical health and social justice viewpoint.

## Data Availability

The raw data supporting the conclusions of this manuscript will be made available by the authors, without undue reservation, to any qualified researcher.

## Author Contributions

SY had full access to all of the data in the study and takes responsibility for the integrity of the data and the accuracy of the data analysis. YZ, YW, HW, GY, and RL contributed substantially to the study design, data analysis and interpretation, and the writing of the manuscript.

### Conflict of Interest Statement

The authors declare that the research was conducted in the absence of any commercial or financial relationships that could be construed as a potential conflict of interest.

## References

[1] Bray F Ferlay J Soerjomataram I Siegel RL Torre LA Jemal A. Global cancer statistics 2018: GLOBOCAN estimates of incidence and mortality worldwide for 36 cancers in 185 countries. CA Cancer J Clin. (2018) 68:394–424. 10.3322/caac.21492 30207593

[2] Siegel R Miller K Jemal A Cancer statistics, 2018. CA Cancer J Clin. (2018) 68:7–30. 10.3322/caac.21442 29313949

[3] Islami F Goding Sauer A Miller KD Siegel RL Fedewa SA Jacobs EJ. Proportion and number of cancer cases and deaths attributable to potentially modifiable risk factors in the United States. CA Cancer J Clin. (2018) 68:31–54. 10.3322/caac.21440 29160902

[4] Moolgavkar SH Holford TR Levy DT Kong CY Foy M Clarke L. Impact of reduced tobacco smoking on lung cancer mortality in the United States during 1975–2000. J Natl Cancer Inst. (2012) 104:541–8. 10.1093/jnci/djs136 22423009 PMC3317881

[5] Houston KA Henley SJ Li J White MC Richards TB. Patterns in lung cancer incidence rates and trends by histologic type in the United States, 2004–2009. Lung Cancer. (2014) 86:22–8. 10.1016/j.lungcan.2014.08.001 25172266 PMC5823254

[6] Lewis DR Check DP Caporaso NE Travis WD Devesa SS. US lung cancer trends by histologic type. Cancer. (2014) 120:2883–92. 10.1002/cncr.28749 25113306 PMC4187244

[7] American Cancer Society Cancer Facts and Figures 2018. Available online at: https://www.cancer.org/research/cancer-facts-statistics/all-cancer-facts-figures/cancer-facts-figures-2018.html (accessed January 6, 2019).

[8] Moyer VA Screening for lung cancer: US preventive services task force recommendation statement. Ann Intern Med. (2014) 160:330–8. 10.7326/M13-2771 24378917

[9] Curran Jr WJ Paulus R Langer CJ Komaki R Lee JS Hauser S. Sequential vs concurrent chemoradiation for stage III non–small cell lung cancer: randomized Phase III trial RTOG 9410. J Natl Cancer Inst. (2011) 103:1452–60. 10.1093/jnci/djr325 21903745 PMC3186782

[10] Bradley JD Paulus R Komaki R Masters G Blumenschein G Schild S. Standard-dose versus high-dose conformal radiotherapy with concurrent and consolidation carboplatin plus paclitaxel with or without cetuximab for patients with stage IIIA or IIIB non-small-cell lung cancer (RTOG 0617): a randomised, two-by-two factorial phase 3 study. Lancet Oncol. (2015) 16:187–99. 10.1016/S1470-2045(14)71207-0 25601342 PMC4419359

[11] Santos FN de Castria TB Cruz MR Riera R Chemotherapy for advanced non-small cell lung cancer in the elderly population. Cochrane Database Syst Rev. (2015) 10:CD010463 10.1002/14651858.CD010463.pub2 PMC6759539 26482542

[12] Davidoff AJ Tang M Seal B Edelman MJ. Chemotherapy and survival benefit in elderly patients with advanced non–small-cell lung cancer. J Clin Oncol. (2010) 28:2191–7. 10.1200/JCO.2009.25.4052 20351329

[13] Meza R Meernik C Jeon J Cote ML. Lung cancer incidence trends by gender, race and histology in the United States, 1973–2010. PLoS ONE. (2015) 10:e0121323. 10.1371/journal.pone.0121323 25822850 PMC4379166

[14] Devesa S Shaw G Blot W. Changing patterns of lung cancer incidence by histological type. Cancer Epidemiol Biomarkers Prev. (1991) 1:29–34. 1845165

[15] Patel MI Cheng I Gomez SL. US lung cancer trends by histologic type. Cancer. (2015) 121:1150–2. 10.1002/cncr.29180 25470142 PMC5746178

[16] Surveillance Epidemiology, End Results (SEER) SEER^*^Stat Database: Incidence - SEER 9 Regs Research Data, Nov 2017 Sub (1973-2015)<Katrina/Rita Population Adjustment> - Linked To County Attributes. Total U.S., 1969-2016 Counties, National Cancer Institute, DCCPS, Surveillance Research Program, released April 2018, based on the November 2017 submission Available online at: www.seer.cancer.gov

[17] Surveillance Epidemiology, End Results (SEER) SEER^*^Stat Database: Mortality - All COD, Aggregated With State, Total U.S. (1969-2015)<Katrina/Rita Population Adjustment>, National Cancer Institute, DCCPS. Surveillance Research Program, released December 2017. Underlying mortality data provided by NCHS. Available online at: www.cdc.gov/nchs.

[18] Chu KC Miller BA Feuer EJ Hankey BF. A method for partitioning cancer mortality trends by factors associated with diagnosis: an application to female breat cancer. J Clin Epidemiol. (1994) 47:1451–61. 10.1016/0895-4356(94)90089-2 7730854

[19] Surveillance Epidemiology, End Results (SEER) SEER^*^Stat Database: Incidence-Based Mortality - SEER 9 Regs Research Data, Nov 2017 Sub (1973-2015)<Katrina/Rita Population Adjustment> - Linked To County Attributes. Total U.S., 1969-2016 Counties, National Cancer Institute, DCCPS, Surveillance Research Program, released April 2018, based on the November 2017 submission. Available online at: www.seer.cancer.gov

[20] National Cancer Institute Localized/Regional/Distant Stage Adjustments. Available online at: https://seer.cancer.gov/seerstat/variables/seer/lrd-stage/ (accessed January 6, 2019).

[21] National Cancer Institute Adjusted AJCC 6th ed T, N, M, and Stage. Available onlien at; https://seer.cancer.gov/seerstat/variables/seer/ajcc-stage/6th/. (accessed January 6, 2019).

[22] National Cancer Institute SEER^*^Stat Software, Latest Release: Version 8.3.5—March 6, 2018. Available online at: https://seer.cancer.gov/seerstat/ (accessed January 6, 2019).

[23] Tiwari RC Clegg LX Zou Z. Efficient interval estimation for age-adjusted cancer rates. Stat Methods Med Res. (2006) 15:547–69. 10.1177/0962280206070621 17260923

[24] National Cancer Institute Joinpoint Trend Analysis Software. Available online at: https://surveillance.cancer.gov/joinpoint/ (accessed January 6, 2019).

[25] Fay MP Tiwari RC Feuer EJ Zou Z. Estimating average annual percent change for disease rates without assuming constant change. Biometrics. (2006) 62:847–54. 10.1111/j.1541-0420.2006.00528.x 16984328

[26] Devesa SS Donaldson J Fears T. Graphical presentation of trends in rates. Am J Epidemiol. (1995) 141:300–4. 10.1093/aje/141.4.300 7840107

[27] Wickham H Tidyverse: easily install and load the ‘Tidyverse'. R package version 1.2. 1. 2017. Available online at: https://cran.r-project.org/web/packages/tidyverse/index.html (accessed January 6, 2019).

[28] R Development Core Team. R: A Language and Environment for Statistical Computing. 2018. Available online at: http://www.R-project.org. (accessed January 6, 2019).

[29] DeVita Jr VT Rosenberg SA. Two hundred years of cancer research. N Engl J Med. (2012) 366:2207–14. 10.1056/NEJMra1204479 22646510 PMC6293471

[30] Thun MJ Carter BD Feskanich D Freedman ND Prentice R Lopez AD. 50-year trends in smoking-related mortality in the United States. N Engl J Med. (2013) 368:351–64. 10.1056/NEJMsa1211127 23343064 PMC3632080

[31] Harris JE Cigarette smoking among successive birth cohorts of men and women in the United States during 1900–1980. J Natl Cancer Inst. (1983) 71:473–9. 6577223

[32] Husten CG Shelton DM Chrismon JH Lin Y Mowery P Powell FA. Cigarette smoking and smoking cessation among older adults: United States, 1965-94. Tob Control. (1997) 6:175–80. 10.1136/tc.6.3.175 9396100 PMC1759574

[33] Jemal A Thun MJ Ries LA Howe HL Weir HK Center MM. Annual report to the nation on the status of cancer, 1975–2005, featuring trends in lung cancer, tobacco use, and tobacco control. J Natl Cancer Inst. (2008) 100:1672–94. 10.1093/jnci/djn389 19033571 PMC2639291

[34] Jeon J Holford TR Levy DT Feuer EJ Cao PP Tam JC. Smoking and lung cancer mortality in the united states from 2015 to 2065: a comparative modeling approach. Ann Intern Med. (2018) 169:684–93. 10.7326/M18-1250 30304504 PMC6242740

[35] Jemal A Cokkinides VE Shafey O Thun MJ. Lung cancer trends in young adults: an early indicator of progress in tobacco control (United States). Cancer Causes Control. (2003) 14:579–85. 10.1023/A:1024891201329 12948289

[36] Jemal A Miller KD Ma J Siegel RL Fedewa SA Islami F. Higher lung cancer incidence in young women than young men in the United States. N Engl J Med. (2018) 378:1999–2009. 10.1056/NEJMoa1715907 29791813 PMC7717174

[37] Jemal A Ma J Rosenberg PS Siegel R Anderson WF. Increasing lung cancer death rates among young women in southern and midwestern States. J Clin Oncol. (2012) 30:2739–44. 10.1200/JCO.2012.42.6098 22734032 PMC3402885

[38] Jemal A Chu KC Tarone RE. Recent trends in lung cancer mortality in the United States. J Natl Cancer Inst. (2001) 93:277–83. 10.1093/jnci/93.4.277 11181774

[39] Jemal A Ward E Thun MJ Contemporary lung cancer trends among US women. Cancer Epidemiol Biomarkers Prev. (2005) 14:582–5. 10.1158/1055-9965.EPI-04-0554 15767333

[40] Pierce JP Lee L Gilpin EA. Smoking initiation by adolescent girls, 1944 through 1988: an association with targeted advertising. JAMA. (1994) 271:608–11. 10.1001/jama.271.8.608 8301793

[41] Holford TR Levy DT Meza R. Comparison of smoking history patterns among African American and white cohorts in the United States born 1890 to 1990. Nicotine Tob Res. (2016) 18:S16–S29. 10.1093/ntr/ntv274 26980861 PMC5009449

[42] Drope J Liber AC Cahn Z Stoklosa M Kennedy R Douglas CE. Who's still smoking? Disparities in adult cigarette smoking prevalence in the United States. CA Cancer J Clin. (2018) 68:106–15. 10.3322/caac.21444 29384589

[43] Gazdar AF Bunn PA Minna JD Small-cell lung cancer: what we know, what we need to know and the path forward. Nat Rev Cancer. (2017) 17:725–37. 10.1038/nrc.2017.87 29077690

[44] Burns DM Anderson CM Gray N. Do changes in cigarette design influence the rise in adenocarcinoma of the lung? Cancer Causes Control. (2011) 22:13–22. 10.1007/s10552-010-9660-0 20967496 PMC3002161

[45] Stepanov I Knezevich A Zhang L Watson CH Hatsukami DK Hecht SS. Carcinogenic tobacco-specific N-nitrosamines in US cigarettes: three decades of remarkable neglect by the tobacco industry. Tob Control. (2012) 21:44–8. 10.1136/tc.2010.042192 21602537 PMC3572908

[46] Devesa SS Bray F Vizcaino AP Parkin DM. International lung cancer trends by histologic type: male: female differences diminishing and adenocarcinoma rates rising. Int J Cancer. (2005) 117:294–9. 10.1002/ijc.21183 15900604

[47] Pesch B Kendzia B Gustavsson P Jöckel KH Johnen G Pohlabeln H. Cigarette smoking and lung cancer—relative risk estimates for the major histological types from a pooled analysis of case–control studies. Int J Cancer. (2012) 131:1210–9. 10.1002/ijc.27339 22052329 PMC3296911

[48] Lubin JH Blot WJ. Assessment of lung cancer risk factors by histologic category. J Natl Cancer Ins. (1984) 73:383–9. 10.1093/jnci/73.2.383 6087006

[49] Gardiner PS. The African Americanization of menthol cigarette use in the United States. Nicotine Tob Res. (2004) 6:S55–S65. 10.1080/14622200310001649478 14982709

[50] Yu D Peterson N Sheffer M Reid R Schnieder J. Tobacco outlet density and demographics: analysing the relationships with a spatial regression approach. Public Health. (2010) 124:412–6. 10.1016/j.puhe.2010.03.024 20541232

[51] Sacher AG Le LW Lau A Earle CC Leighl NB. Real-world chemotherapy treatment patterns in metastatic non–small cell lung cancer: are patients undertreated? Cancer. (2015) 121:2562–9. 10.1002/cncr.29386 25891153

[52] Schwartz S. The fallacy of the ecological fallacy: the potential misuse of a concept and the consequences. Am J Public Health. (1994) 84:819–24. 10.2105/AJPH.84.5.819 8179055 PMC1615039

[53] German RR1 Fink AK Heron M Stewart SL Johnson CJ Finch JL. The accuracy of cancer mortality statistics based on death certificates in the United States. Cancer Epidemiol. (2011) 35:126–31. 10.1016/j.canep.2010.09.005 20952269

