# Supplementary material for: Trends and Patterns of Disparities in Burden of Lung Cancer in the United States, 1974-2015
Source: Front Oncol. 2019 May 31;9:404. doi: 10.3389/fonc.2019.00404 (PMC6555199; doi:10.3389/fonc.2019.00404)
Supplement: Supplementary file 1 [file Data_Sheet_1.docx]

**Supplementary Materials**

Supplementary Table 1. Trends in Lung Cancer Incidence Rates (1974-2015)

Supplementary Table 2. Trends in Lung Cancer Mortality Rates (1989-2015)

Supplementary Table 3. Trends in Lung Cancer Incidence (1974-2015) and Mortality (1989-2015) Rates Among 20-39 Year-Olds According to Sex

Supplementary Table 4. Trends in Lung Cancer Incidence Rates According to Histologic Type, Race, and Sex (1974-2015)

Supplementary Table 5. Trends in Lung Cancer Incidence (1988-2015) and Mortality (1989-2015) Rates among Elderly Aged 80 and above by SEER Stage

Supplementary Table 6. Trends in Lung Cancer Incidence (2004-2015) and Mortality (2004-2015) Rates among Elderly Aged 80 and above by TNM Stage

Supplementary Figure 1. Trends in Overall Lung Cancer Incidence Rates by Sex (1974-2015)

Supplementary Figure 2. Trends in Overall Lung Cancer Mortality Rates by Sex (1989-2015)

Supplementary Figure 3. Trends in Small Cell Carcinoma Incidence Rates by Sex (1974-2015)

Supplementary Figure 4. Trends in Small Cell Carcinoma Mortality Rates by Sex (1989-2015)

Supplementary Figure 5. Trends in Adenocarcinoma Incidence Rates by Sex (1974-2015)

Supplementary Figure 6. Trends in Adenocarcinoma Mortality Rates by Sex (1989-2015)

Supplementary Figure 7. Trends in Squamous Cell Carcinoma Incidence Rates by Sex (1974-2015)

Supplementary Figure 8. Trends in Squamous Cell Carcinoma Mortality Rates by Sex (1989-2015)

Supplementary Figure 9. Trends in Overall Lung Cancer Incidence Rates by Race (1974-2015)

Supplementary Figure 10. Trends in Overall Lung Cancer Mortality Rates by Race (1989-2015)

Supplementary Figure 11. Trends in Small Cell Carcinoma Incidence Rates by Race (1974-2015)

Supplementary Figure 12. Trends in Small Cell Carcinoma Mortality Rates by Race (1989-2015)

Supplementary Figure 13. Trends in Adenocarcinoma Incidence Rates by Race (1974-2015)

Supplementary Figure 14. Trends in Adenocarcinoma Mortality Rates by Race (1989-2015)

Supplementary Figure 15. Trends in Squamous Cell Carcinoma Incidence Rates by Race (1974-2015)

Supplementary Figure 16. Trends in Squamous Cell Carcinoma Mortality Rates by Race (1989-2015)

Supplementary Figure 17. Trends in Overall Lung Cancer Incidence Rates by Age at Diagnosis (1974-2015)

Supplementary Figure 18. Trends in Overall Lung Cancer Mortality Rates by Age at Diagnosis (1989-2015)

Supplementary Figure 19. Trends in Small Cell Carcinoma Incidence Rates by Age at Diagnosis (1974-2015)

Supplementary Figure 20. Trends in Small Cell Carcinoma Mortality Rates by Age at Diagnosis (1989-2015)

Supplementary Figure 21. Trends in Adenocarcinoma Incidence Rates by Age at Diagnosis (1974-2015)

Supplementary Figure 22. Trends in Adenocarcinoma Mortality Rates by Age at Diagnosis (1989-2015)

Supplementary Figure 23. Trends in Squamous Cell Carcinoma Incidence Rates by Age at Diagnosis (1974-2015)

Supplementary Figure 24. Trends in Squamous Cell Carcinoma Mortality Rates by Age at Diagnosis (1989-2015)

Supplementary Figure 25. Trends in Overall Lung Cancer Incidence Rates by SEER Stage at Diagnosis (1988-2015)

Supplementary Figure 26. Trends in Overall Lung Cancer Mortality Rates by SEER Stage at Diagnosis (1989-2015)

Supplementary Figure 27. Trends in Small Cell Carcinoma Incidence Rates by SEER Stage at Diagnosis (1988-2015)

Supplementary Figure 28. Trends in Small Cell Carcinoma Mortality Rates by SEER Stage at Diagnosis (1989-2015)

Supplementary Figure 29. Trends in Adenocarcinoma Incidence Rates by SEER Stage at Diagnosis (1988-2015)

Supplementary Figure 30. Trends in Adenocarcinoma Mortality Rates by SEER Stage at Diagnosis (1989-2015)

Supplementary Figure 31. Trends in Squamous Cell Carcinoma Incidence Rates by SEER Stage at Diagnosis (1988-2015)

Supplementary Figure 32. Trends in Squamous Cell Carcinoma Mortality Rates by SEER Stage at Diagnosis (1989-2015)

Supplementary Figure 33. Trends in Overall Lung Cancer Incidence Rates by TNM Stage at Diagnosis (2004-2015)

Supplementary Figure 34. Trends in Overall Lung Cancer Mortality Rates by TNM Stage at Diagnosis (2004-2015)

Supplementary Figure 35. Trends in Small Cell Carcinoma Incidence Rates by TNM Stage at Diagnosis (2004-2015)

Supplementary Figure 36. Trends in Small Cell Carcinoma Mortality Rates by TNM Stage at Diagnosis (2004-2015)

Supplementary Figure 37. Trends in Adenocarcinoma Incidence Rates by TNM Stage at Diagnosis (2004-2015)

Supplementary Figure 38. Trends in Adenocarcinoma Mortality Rates by TNM Stage at Diagnosis (2004-2015)

Supplementary Figure 39. Trends in Squamous Cell Carcinoma Incidence Rates by TNM Stage at Diagnosis (2004-2015)

Supplementary Figure 40. Trends in Squamous Cell Carcinoma Mortality Rates by TNM Stage at Diagnosis (2004-2015)

Supplementary Table 1. Trends in Lung Cancer Incidence Rates (1974-2015)^a^

| **Histologic type** | **Overall (1974-2015)** | | **Trend^b^** | | | | | | | | | | | | | | | | |
| --- | --- | --- | --- | --- | --- | --- | --- | --- | --- | --- | --- | --- | --- | --- | --- | --- | --- | --- | --- |
|  |  |  | **1** | | | **2** | | | | **3** | | | | **4** | | | **5** | | |
|  | **AAPC**  **(95% CI)** | ***P* Value** | **Year** | **APC**  **(95% CI)** | ***P* Value** | **Year** | **APC**  **(95% CI)** | ***P* Value** | **Year** | | **APC**  **(95% CI)** | ***P* Value** | **Year** | | **APC**  **(95% CI)** | ***P* Value** | **Year** | **APC**  **(95% CI)** | ***P* Value** |
| Overall | 0.0  (-0.1 to 0.1) | 0.939 | 1974-1981 | 3.8  (3.4 to 4.3) | <0.001 | 1981-1990 | 1.1  (0.7 to 1.4) | <0.001 | 1990-2007 | | -0.9  (-1.0 to -0.8) | <0.001 | 2007-2015 | | -2.6  (-2.9 to -2.2) | <0.001 |  |  |  |
| Small cell carcinoma | -0.1  (-0.4 to 0.1) | 0.361 | 1974-1981 | 7.1  (6.0 to 8.2) | <0.001 | 1981-1989 | 1.8  (0.7 to 2.8) | 0.001 | 1989-2015 | | -2.5  (-2.7 to -2.4) | <0.001 |  | |  |  |  |  |  |
| Adeno-carcinoma | 2.0  (1.6 to 2.5) | <0.001 | 1974-1978 | 10.1  (7.5 to 12.7) | <0.001 | 1978-1992 | 2.9  (2.5 to 3.3) | <0.001 | 1992-2005 | | -0.8  (-1.2 to -0.4) | 0.001 | 2005-2011 | | 2.7  (1.0 to 4.4) | 0.003 | 2011-2015 | -0.4 (-2.7 to 2.0) | 0.722 |
| Squamous cell carcinoma | -1.0  (-1.4 to -0.6) | <0.001 | 1974-1982 | 2.4  (1.7 to 3.2) | <0.001 | 1982-1991 | -1.2  (-1.9 to -0.5) | 0.002 | 1991-2005 | | -3.2  (-3.5 to -2.8) | <0.001 | 2005-2010 | | 1.5  (-0.6 to 3.6) | 0.165 | 2010-2015 | -2.3 (-3.7 to -0.8) | 0.003 |

Abbreviations: AAPC, average annual percent change; APC, annual percent change.

^a^ Rates were calculated as number of cases per 100000 person-years and age adjusted to the 2000 US standard population.

^b^ The calendar period of each segment was defined based on the identification of calendar years when a statistically significant change in the APC occurred (ie. the joinpoint).

Supplementary Table 2. Trends in Lung Cancer Mortality Rates (1989-2015)^a^

| **Histologic type** | **Overall (1989-2015)** | | | **Trend^b^** | | | | | | | | | | | | | |
| --- | --- | --- | --- | --- | --- | --- | --- | --- | --- | --- | --- | --- | --- | --- | --- | --- | --- |
|  |  |  |  | **1** | | | **2** | | | | **3** | | | **4** | | | |
|  | **AAPC**  **(95% CI)** | ***P* Value** | **Year** | | **APC**  **(95% CI)** | ***P* Value** | **Year** | **APC**  **(95% CI)** | ***P* Value** | **Year** | | **APC**  **(95% CI)** | ***P* Value** | **Year** | **APC**  **(95% CI)** | ***P* Value** |  |
| Overall | -1.7  (-1.9 to -1.4) | <0.001 | 1989-1991 | | 1.0  (-1.5 to 3.6) | 0.397 | 1991-2004 | -1.1  (-1.2 to -0.9) | <0.001 | 2004-2010 | | -2.3  (-2.8 to -1.7) | <0.001 | 2010-2015 | -3.6  (-4.2 to -3.1) | <0.001 |  |
| Small cell carcinoma | -2.6  (-2.8 to -2.4) | <0.001 | 1989-2001 | | -2.0  (-2.4 to -1.6) | <0.001 | 2001-2015 | -3.1  (-3.4 to -2.8) | <0.001 |  | |  |  |  |  |  |  |
| Adeno-carcinoma | 0.0  (-0.5 to 0.6) | 0.908 | 1989-1996 | | 1.2  (0.5 to 2.0) | 0.004 | 1996-2006 | -1.6  (-2.1 to -1.1) | <0.001 | 2006-2013 | | 2.0  (1.0 to 3.0) | <0.001 | 2013-2015 | -2.8  (-8.3 to 3.0) | 0.312 |  |
| Squamous cell carcinoma | -2.6  (-3.3 to -1.9) | <0.001 | 1989-2007 | | -3.2  (-3.5 to -2.9) | <0.001 | 2007-2012 | 1.1  (-1.8 to 4.0) | 0.434 | 2012-2015 | | -5.0  (-9.2 to -0.5) | 0.030 |  |  |  |  |

Abbreviations: AAPC, average annual percent change; APC, annual percent change.

^a^ Rates were calculated as number of cases per 100000 person-years and age adjusted to the 2000 US standard population.

^b^ The calendar period of each segment was defined based on the identification of calendar years when a statistically significant change in the APC occurred (ie. the joinpoint).

Supplementary Table 3. Trends in Lung Cancer Incidence (1974-2015) and Mortality (1989-2015) Rates Among 20-39 Year-Olds According to Sex^a^

| **Indicator** | **Gender** | **Overall** | | **Trend^b^** | | | | | |
| --- | --- | --- | --- | --- | --- | --- | --- | --- | --- |
|  |  |  |  | **1** | | | **2** | | |
|  |  | **AAPC (95% CI)** | ***P* Value** | **Year** | **APC (95% CI)** | ***P* Value** | **Year** | **APC (95% CI)** | ***P* Value** |
| Incidence (1974-2015) |  |  |  |  |  |  |  |  |  |
|  | Male | -2.5 (-2.8, -2.2) | <0.001 | 1974-2015 | -2.5 (-2.8, -2.2) | <0.001 |  |  |  |
|  | Female | -1.7 (-2.5, -1.0) | <0.001 | 1974-1999 | -0.8 (-1.5, -0.1) | 0.033 | 1999-2015 | -3.1 (-4.7, -1.5) | <0.001 |
| Mortality (1989-2015) |  |  |  |  |  |  |  |  |  |
|  | Male | -3.4 (-4.1, -2.6) | <0.001 | 1989-2015 | -3.4 (-4.1, -2.6) | <0.001 |  |  |  |
|  | Female | -2.5 (-4.4, -0.6) | 0.010 | 1989-1997 | 3.3 (-2.3, 9.2) | 0.239 | 1997-2015 | -5.0 (-6.5, -3.4) | <0.001 |

Abbreviations: AAPC, average annual percent change; APC, annual percent change.

a Rates were calculated as number of cases per 100000 person-years and age adjusted to the 2000 US standard population.

b The calendar period of each segment was defined based on the identification of calendar years when a statistically significant change in the APC occurred (ie. the joinpoint).

Supplementary Table 4. Trends in Lung Cancer Incidence Rates According to Histologic Type, Race, and Sex (1974-2015)^a^

| **Histologic type** | **Race by gender** | **Overall** | | **Trend^b^** | | | | | | | | | | | | | | | | | |
| --- | --- | --- | --- | --- | --- | --- | --- | --- | --- | --- | --- | --- | --- | --- | --- | --- | --- | --- | --- | --- | --- |
|  |  |  |  | **1** | | | **2** | | | **3** | | | **4** | | | **5** | | | **6** | | |
|  |  | **AAPC**  **(95% CI)** | ***P* Value** | **Year** | **APC**  **(95% CI)** | ***P* Value** | **Year** | **APC**  **(95% CI)** | ***P* Value** | **Year** | **APC**  **(95% CI)** | ***P* Value** | **Year** | **APC**  **(95% CI)** | ***P* Value** | **Year** | **APC**  **(95% CI)** | ***P* Value** | **Year** | **APC**  **(95% CI)** | ***P* Value** |
| Small cell carcinoma |  |  |  |  |  |  |  |  |  |  |  |  |  |  |  |  |  |  |  |  |  |
|  | Black male | -1.0  (-1.7, -0.4) | 0.001 | 1974-1985 | 6.0  (3.9, 8.3) | <0.001 | 1985-2015 | -3.5  (-4.0, -3.1) | <0.001 |  |  |  |  |  |  |  |  |  |  |  |  |
|  | Black female | 0.6  (-0.2, 1.5) | 0.140 | 1975^c^-1991 | 4.9  (3.2, 6.7) | <0.001 | 1991-2015 | -2.1  (-3.0, -1.2) | <0.001 |  |  |  |  |  |  |  |  |  |  |  |  |
|  | White male | -1.0  (-1.4, -0.6) | <0.001 | 1974-1978 | 8.4  (5.0 12.0) | <0.001 | 1978-1986 | 2.2  (0.8, 3.6) | 0.003 | 1986-2015 | -3.1  (-3.2, -2.9) | <0.001 |  |  |  |  |  |  |  |  |  |
|  | White female | 1.5  (1.1, 2.0) | <0.001 | 1974-1982 | 10.1  (8.6, 11.6) | <0.001 | 1982-1991 | 3.4  (1.9, 4.8) | <0.001 | 1991-2015 | -1.8  (-2.1, -1.5) | <0.001 |  |  |  |  |  |  |  |  |  |
| Adeno-carcinoma | Black male | 1.8  (0.7, 2.9) | 0.001 | 1974-1978 | 16.5  (7.8, 25.8) | <0.001 | 1978-1994 | 1.6  (0.5, 2.6) | 0.005 | 1994-2004 | -2.9  (-5.0, -0.7) | 0.011 | 2004-2015 | 1.5  (-0.1, 3.2) | 0.065 |  |  |  |  |  |  |
|  | Black female | 3.1  (2.1, 4.2) | <0.001 | 1974-1979 | 12.9  (8.5, 17.4) | <0.001 | 1979-1991 | 3.9  (2.7, 5.1) | <0.001 | 1991-2005 | 0.3  (-0.7, 1.2) | 0.581 | 2005-2012 | 4.6  (1.6, 7.8) | 0.004 | 2012-2015 | -5.0  (-13.0, 3.7) | 0.242 |  |  |  |
|  | White male | 1.3  (0.9, 1.7) | <0.001 | 1974-1979 | 7.9  (5.6, 10.2) | <0.001 | 1979-1992 | 1.9  (1.4, 2.5) | <0.001 | 1992-2005 | -1.6  (-2.2, -1.0) | <0.001 | 2005-2015 | 1.0  (0.3, 1.8) | 0.011 |  |  |  |  |  |  |
|  | White female | 3.2  (2.4, 3.9) | <0.001 | 1974-1976 | 18.0  (8.8, 28.0) | <0.001 | 1976-1988 | 5.1  (4.6, 5.7) | <0.001 | 1988-1998 | 1.8  (1.0, 2.6) | <0.001 | 1998-2001 | -2.6  (-10.2, 5.7) | 0.515 | 2001-2015 | 1.7  (1.4, 2.1) | <0.001 |  |  |  |
| Squamous cell carcinoma | Black male | -2.4  (-2.8, -1.9) | <0.001 | 1974-1984 | 1.8  (0.1, 3.6) | 0.037 | 1984-2015 | -3.7  (-4.0, -3.4) | <0.001 |  |  |  |  |  |  |  |  |  |  |  |  |

| Supplementary Table 4. Trends in Lung Cancer Incidence Rates According to Histologic Type, Race, and Sex^a^ (continued) | | | | | | | | | | | | | | | | | | | | | |
| --- | --- | --- | --- | --- | --- | --- | --- | --- | --- | --- | --- | --- | --- | --- | --- | --- | --- | --- | --- | --- | --- |
| **Histologic type** | **Race by gender** | **Overall** | | **Trend^b^** | | | | | | | | | | | | | | | | | |
|  |  |  |  | **1** | | | **2** | | | **3** | | | **4** | | | **5** | | | **6** | | |
|  |  | **AAPC**  **(95% CI)** | ***P* Value** | **Year** | **APC**  **(95% CI)** | ***P* Value** | **Year** | **APC**  **(95% CI)** | ***P* Value** | **Year** | **APC**  **(95% CI)** | ***P* Value** | **Year** | **APC**  **(95% CI)** | ***P* Value** | **Year** | **APC**  **(95% CI)** | ***P* Value** | **Year** | **APC**  **(95% CI)** | ***P* Value** |
|  | Black female | 1.0  (0.2, 1.8) | 0.017 | 1974-1986 | 6.0  (3.5, 8.5) | <0.001 | 1986-2015 | -1.1  (-1.7, -0.4) | 0.001 |  |  |  |  |  |  |  |  |  |  |  |  |
|  | White male | -1.8  (-2.3, -1.2) | <0.001 | 1974-1981 | 2.2  (1.0, 3.4) | 0.001 | 1981-1989 | -1.8  (-3.0, -0.6) | 0.004 | 1989-2005 | -4.0  (-4.3, -3.6) | <0.001 | 2005-2010 | 1.4  (-1.4, 4.2) | 0.330 | 2010-2015 | -3.0  (-4.9, -1.0) | 0.004 |  |  |  |
|  | White female | 1.5  (1.0, 2.0) | <0.001 | 1974-1979 | 8.2  (6.4, 9.9) | <0.001 | 1979-1986 | 4.1  (2.8, 5.4) | <0.001 | 1986-1996 | 0.7  (0.0, 1.3) | 0.055 | 1996-2004 | -2.5  (-3.5, -1.6) | <0.001 | 2004-2010 | 2.8  (1.2, 4.5) | 0.002 | 2010-2015 | -1.8  (-3.4, -0.2) | 0.028 |

Abbreviations: AAPC, average annual percent change; APC, annual percent change.

^a^ Rates were calculated as number of cases per 100000 person-years and age adjusted to the 2000 US standard population.

^b^ The calendar period of each segment was defined based on the identification of calendar years when a statistically significant change in the APC occurred (ie. the joinpoint).

Supplementary Table 5. Trends in Lung Cancer Incidence (1988-2015) and Mortality (1989-2015) Rates among Elderly Aged 80 and above by SEER Stage*^a^*

| **Indicator** | **SEER Stage** | **Overall** | | **Trend^b^** | | | | | | | | | | | | | | | | |
| --- | --- | --- | --- | --- | --- | --- | --- | --- | --- | --- | --- | --- | --- | --- | --- | --- | --- | --- | --- | --- |
|  |  |  |  | **1** | | | **2** | | | **3** | | | **4** | | | | **5** | | | |
|  |  | **AAPC**  **(95% CI)** | ***P* Value** | **Year** | **APC**  **(95% CI)** | ***P* Value** | **Year** | **APC**  **(95% CI)** | ***P* Value** | **Year** | **APC**  **(95% CI)** | ***P* Value** | **Year** | **APC**  **(95% CI)** | **P Value** | **Year** | | **APC**  **(95% CI)** | ***P* Value** |  |
| Incidence |  |  |  |  |  |  |  |  |  |  |  |  |  |  |  |  | |  |  |  |
|  | Localized | 2.6  (1.9, 3.2) | <0.001 | 1988-2009 | 3.5  (3.1, 3.9) | <0.001 | 2009-2015 | -0.5  (-3.1, 2.1) | 0.678 |  |  |  |  |  |  |  | |  |  |  |
|  | Regional | 1.1  (-0.1, 2.3) | 0.069 | 1988-1997 | 4.7  (3.0, 6.4) | <0.001 | 1997-2012 | 0.8  (-0.1, 1.6) | 0.074 | 2012-2015 | -7.5  (-15.6, 1.3) | 0.087 |  |  |  |  | |  |  |  |
|  | Distant | 1.5  (0.9, 2.1) | <0.001 | 1988-2000 | 2.2  (1.5, 2.8) | <0.001 | 2000-2007 | 4.4  (2.6, 6.3) | <0.001 | 2007-2015 | -1.9  (-3.0, -0.8) | 0.002 |  |  |  |  | |  |  |  |
| Mortality |  |  |  |  |  |  |  |  |  |  |  |  |  |  |  |  | |  |  |  |
|  | Localized | 4.3  (3.3, 5.2) | <0.001 | 1989-1996 | 12.6  (9.1, 16.2) | <0.001 | 1996-2015 | 1.4  (0.6, 2.1) | 0.001 |  |  |  |  |  |  |  | |  |  |  |
|  | Regional | 2.8  (1.0, 4.6) | 0.002 | 1989-1991 | 22.8  (7.8, 40.0) | 0.005 | 1991-1995 | 8.1  (1.3, 15.4) | 0.023 | 1995-2000 | 2.5  (-1.7, 6.8) | 0.227 | 2000-2013 | -0.1  (-0.8, 0.7) | 0.886 | 2013-2015 | | -6.1  (-17.6, 7.0) | 0.319 |  |
|  | Distant | 1.7  (1.2, 2.2) | <0.001 | 1989-2009 | 2.8  (2.5, 3.1) | <0.001 | 2009-2015 | 1.7  (1.2, 2.2) | <0.001 |  |  |  |  |  |  |  | |  |  |  |

Abbreviations: AAPC, average annual percent change; APC, annual percent change.

^a^ Rates were calculated as number of cases per 100000 person-years and age adjusted to the 2000 US standard population.

^b^ The calendar period of each segment was defined based on the identification of calendar years when a statistically significant change in the APC occurred (ie. the joinpoint).

Supplementary Table 6. Trends in Lung Cancer Incidence (2004-2015) and Mortality (2004-2015) Rates among Elderly Aged 80 and above by TNM Stage^a^

| **Indicator** | **TNM Stage** | **Overall** | | | **Trend^b^** | | | | | | | |
| --- | --- | --- | --- | --- | --- | --- | --- | --- | --- | --- | --- | --- |
|  |  |  |  |  | **1** | | | | **2** | | | |
|  |  | **AAPC (95% CI)** | **P Value** | **Year** | | **APC (95% CI)** | **P Value** | **Year** | | **APC (95% CI)** | **P Value** |  |
| Incidence |  |  |  |  | |  |  |  | |  |  |  |
|  | I | 3.1 (2.2, 4.1) | <0.001 | 2004-2009 | | 6.9 (5.0, 8.8) | <0.001 | 2009-2015 | | 0.1 (-1.3, 1.4) | 0.911 |  |
|  | II | -9.8 (-3.5, 1.6) | 0.415 | 2004-2015 | | -9.8 (-3.5, 1.6) | 0.415 |  | |  |  |  |
|  | III | -1.5 (-3.4, 0.5) | 0.136 | 2004-2010 | | 1.1 (-1.8, 4.1) | 0.400 | 2010-2015 | | -4.5 (-8.2, -0.8) | 0.026 |  |
|  | IV | 0.7 (-0.6, 1.9) | 0.300 | 2004-2010 | | 2.7 (0.9, 4.7) | 0.011 | 2010-2015 | | -1.7 (-4.1, 0.7) | 0.134 |  |
| Mortality |  |  |  |  | |  |  |  | |  |  |  |
|  | I | 21.4 (16.9, 26.1) | <0.001 | 2004-2006 | | 116.9 (71.7, 174.0) | <0.001 | 2006-2015 | | 6.7 (4.5, 9.0) | <0.001 |  |
|  | II | 1.5 (-1.7, 4.8) | 0.332 | 2005^c^-2015 | | 1.5 (-1.7, 4.8) | 0.332 |  | |  |  |  |
|  | III | 6.6 (4.2, 9.1) | <0.001 | 2004-2006 | | 46.0 (26.9, 67.9) | <0.001 | 2006-2015 | | -0.6 (-1.9, 0.7) | 0.310 |  |
|  | IV | 5.5 (2.6, 8.4) | <0.001 | 2004-2006 | | 32.0 (11.5, 56.3) | 0.006 | 2006-2015 | | 0.4 (-1.2, 1.9) | 0.580 |  |

Abbreviations: AAPC, average annual percent change; APC, annual percent change.

^a^ Rates were calculated as number of cases per 100000 person-years and age adjusted to the 2000 US standard population.

^b^ The calendar period each segment was defined based on the identification of calendar years when a statistically significant change in the APC occurred (ie. the joinpoint).

^c^ Trends for mortality of TNM stage II were calculated starting from the year 2005 due to suppressed statistic because of fewer than 16 deaths in 2004.

Supplementary Figure 1. Trends in Overall Lung Cancer Incidence Rates by Sex (1974-2015)

Supplementary Figure 2. Trends in Overall Lung Cancer Mortality Rates by Sex (1989-2015)

Supplementary Figure 3. Trends in Small Cell Carcinoma Incidence Rates by Sex (1974-2015)

Supplementary Figure 4. Trends in Small Cell Carcinoma Mortality Rates by Sex (1989-2015)

Supplementary Figure 5. Trends in Adenocarcinoma Incidence Rates by Sex (1974-2015)

Supplementary Figure 6. Trends in Adenocarcinoma Mortality Rates by Sex (1989-2015)

Supplementary Figure 7. Trends in Squamous Cell Carcinoma Incidence Rates by Sex (1974-2015)

Supplementary Figure 8. Trends in Squamous Cell Carcinoma Mortality Rates by Sex (1989-2015)

Supplementary Figure 9. Trends in Overall Lung Cancer Incidence Rates by Race (1974-2015)

Supplementary Figure 10. Trends in Overall Lung Cancer Mortality Rates by Race (1989-2015)

Supplementary Figure 11. Trends in Small Cell Carcinoma Incidence Rates by Race (1974-2015)

Supplementary Figure 12. Trends in Small Cell Carcinoma Mortality Rates by Race (1989-2015)

Supplementary Figure 13. Trends in Adenocarcinoma Incidence Rates by Race (1974-2015)

Supplementary Figure 14. Trends in Adenocarcinoma Mortality Rates by Race (1989-2015)

Supplementary Figure 15. Trends in Squamous Cell Carcinoma Incidence Rates by Race (1974-2015)

Supplementary Figure 16. Trends in Squamous Cell Carcinoma Mortality Rates by Race (1989-2015)

Supplementary Figure 17. Trends in Overall Lung Cancer Incidence Rates by Age at Diagnosis (1974-2015)

Supplementary Figure 18. Trends in Overall Lung Cancer Mortality Rates by Age at Diagnosis (1989-2015)

Supplementary Figure 19. Trends in Small Cell Carcinoma Incidence Rates by Age at Diagnosis (1974-2015)

Supplementary Figure 20. Trends in Small Cell Carcinoma Mortality Rates by Age at Diagnosis (1989-2015)

Supplementary Figure 21. Trends in Adenocarcinoma Incidence Rates by Age at Diagnosis (1988-2015)

Supplementary Figure 22. Trends in Adenocarcinoma Mortality Rates by Age at Diagnosis (1989-2015)

Supplementary Figure 23. Trends in Squamous Cell Carcinoma Incidence Rates by Age at Diagnosis (1974-2015)

Supplementary Figure 24. Trends in Squamous Cell Carcinoma Mortality Rates by Age at Diagnosis (1989-2015)

Supplementary Figure 25. Trends in Overall Lung Cancer Incidence Rates by SEER Stage at Diagnosis (1988-2015)

Supplementary Figure 26. Trends in Overall Lung Cancer Mortality Rates by SEER Stage at Diagnosis (1989-2015)

Supplementary Figure 27. Trends in Small Cell Carcinoma Incidence Rates by SEER Stage at Diagnosis (1988-2015)

Supplementary Figure 28. Trends in Small Cell Carcinoma Mortality Rates by SEER Stage at Diagnosis (1989-2015)

Supplementary Figure 29. Trends in Adenocarcinoma Incidence Rates by SEER Stage at Diagnosis (1988-2015)

Supplementary Figure 30. Trends in Adenocarcinoma Mortality Rates by SEER Stage at Diagnosis (1989-2015)

Supplementary Figure 31. Trends in Squamous Cell Carcinoma Incidence Rates by SEER Stage at Diagnosis (1988-2015)

Supplementary Figure 32. Trends in Squamous Cell Carcinoma Mortality Rates by SEER Stage at Diagnosis (1989-2015)

Supplementary Figure 33. Trends in Overall Lung Cancer Incidence Rates by TNM Stage at Diagnosis (2004-2015)

Supplementary Figure 34. Trends in Overall Lung Cancer Mortality Rates by TNM Stage at Diagnosis (2004-2015)

Supplementary Figure 35. Trends in Small Cell Carcinoma Incidence Rates by TNM Stage at Diagnosis (2004-2015)

Supplementary Figure 36. Trends in Small Cell Carcinoma Mortality Rates by TNM Stage at Diagnosis (2004-2015)

Supplementary Figure 37. Trends in Adenocarcinoma Incidence Rates by TNM Stage at Diagnosis (2004-2015)

Supplementary Figure 38. Trends in Adenocarcinoma Mortality Rates by TNM Stage at Diagnosis (2004-2015)

Supplementary Figure 39. Trends in Squamous Cell Carcinoma Incidence Rates by TNM Stage at Diagnosis (2004-2015)

Supplementary Figure 40. Trends in Squamous Cell Carcinoma Mortality Rates by TNM Stage at Diagnosis (2004-2015)
